# Preparation of antioxidant peptides from *Moringa oleifera* leaves and their protection against oxidative damage in HepG2 cells

**DOI:** 10.3389/fnut.2022.1062671

**Published:** 2022-12-01

**Authors:** Liang Tao, Fan Gu, Yan Liu, Min Yang, Xing-Zhong Wu, Jun Sheng, Yang Tian

**Affiliations:** ^1^College of Food Science and Technology, Yunnan Agricultural University, Kunming, China; ^2^National Research and Development Professional Center for Moringa Processing Technology, Yunnan Agricultural University, Kunming, China; ^3^Engineering Research Center of Development and Utilization of Food and Drug Homologous Resources, Ministry of Education, Yunnan Agricultural University, Kunming, China; ^4^Yunnan Provincial Engineering Research Center for Edible and Medicinal Homologous Functional Food, Yunnan Agricultural University, Kunming, China

**Keywords:** *Moringa oleifera* leaves, antioxidant peptide, preparation, antioxidant activity, apoptosis

## Abstract

*Moringa oleifera* leaves are a kind of new food raw materials, rich in functional factors, *M. oleifera* leaves aqueous extract have antioxidant activity and *M. oleifera* leave protein is an important active ingredient in the aqueous extract. Numerous studies have shown that peptides have strong antioxidant activity. To reveal the antioxidant effects of *M. oleifera* (MO) leaves peptides, MO leave antioxidant peptides were isolated and prepared to clarify their antioxidant activity. MLPH1 (<1 kDa), MLPH3 (1~3 kDa), MLPH5 (3~5 kDa), and MLPH10 (5~10 kDa) fractions were obtained by the membrane ultrafiltration classification of MO leaves proteolytic hydrolysate (MLPH). MLPH1 was further separated by centrifugal filters, and the fraction separated by <1 kDa (MLPH1-1) was identified and analyzed by LC–MS/MS. The purpose of this study was to investigate the effect of MO leaves antioxidant peptide pretreatment on H_2_O_2_-treated HepG2 cells and to refine the antioxidant activity. The results showed that MLPH1 had the strongest antioxidant activity, and three MO leaves antioxidant peptides (LALPVYN, LHIAALVFQ, and FHEEDDAKLF) were obtained. The peptide with the sequence LALPVYN and a molecular weight of 788.44 Da had the strongest antioxidant activity. After 24 h of LALPVYN pretreatment, the cell viability and the CAT, GSH-Px, and SOD enzyme activity were significantly increased, and the MDA, ROS, and apoptosis rates were significantly decreased. These results provide a theoretical basis for further research on the antioxidant mechanism of MO leaves peptides.

## Introduction

Redox reactions are relatively common life activities that occur in living organisms (1). During cellular metabolism, reactive oxygen species (ROS) are generated, including O^2−^ (superoxide anion radical), –OH (hydroxyl radical), and H_2_O_2_ (hydrogen peroxide). In these normal conditions, excess ROS is scavenged by endogenous antioxidant defense systems, including enzymatic antioxidant systems [superoxide dismutase (SOD), catalase (CAT), glutathione (GSH) etc.] and non-enzymatic antioxidant systems in the body (glutamate, ascorbic acid, tocopherol, carnosine, etc.). In addition, low concentrations of ROS mainly act as growth factors and intercellular signaling molecules (2). However, when the antioxidant capacity of the body is imbalanced, the imbalance of ROS production and scavenging can lead to an excessive accumulation of ROS, which can cause oxidative stress damage to cells and tissues (3). When this damage is not repaired in a timely manner and accumulates, it can lead to various diseases, such as cancer, cardiovascular disease, and inflammatory diseases (4, 5). This is where supplementation with natural antioxidants is crucial. Natural antioxidants (peptides, polysaccharides, et al.) are not only popular (6), but also maintain the redox dynamic balance of the body (7).

*Moringa oleifera* (MO) belongs to the genus *Moringa* in the family Moraceae and is a perennial tropical deciduous tree plant, with ~14 species worldwide, which is rich in nutrients and available in its entirety. MO is highly adaptable to soil conditions and rainfall, grows rapidly, and is widely grown in tropical and subtropical regions of Asia and Africa. MO leaves are approved as a new resource food with high nutritional value and various biological activities, such as antibacterial activity (8, 9), anti-inflammatory activity (10), and antioxidant activity (11–13). The protein content of MO leaves can reach up to 34%, which is ~10 times higher than that of milk (3.3%). The rich protein content is a good source of peptides, and the study showed that MO leaves aqueous extract has antioxidant activity (14, 15). In this study, we found that the clearance of DPPH was 79.22% at a concentration of 23.3 mg/g by alkaline protease hydrolysis of MO leaves proteolytic hydrolysates (MLPH). However, the main active components in MLPH and their protective effects on cellular oxidative damage still need to be further investigated. A large number of studies have shown that natural peptides have good antioxidant activity, with the advantages of safety and non-toxic side effects (16). Liang et al. (17) used HepG2 cells as a model of oxidative damage to study the effects of pulsed electric field (PEF) treatment on the intracellular antioxidant and apoptotic activities of the peptide Lys-Asp-His-Cys-His (KDHCH). Wang et al. (1) used HepG2 cells as a model of oxidative damage to investigate the ability of corn gluten peptide fractions (CPFs < 1 kDa) to scavenge intracellular reactive oxygen species (ROS) and regulate antioxidant enzymes in HepG2 cells. Therefore, it is important to study the antioxidant properties of peptides in MO leaves. These studies will act as a guide for developing food or medicine.

At present, antioxidant research on MO leaves in various countries mainly focuses on aqueous extracts. Soliman et al. (14) demonstrated the protective effects of MO leaves extract against oxidative stress and hepatic and renal injuries caused by methotrexate (MTX) therapy. Kumar et al. (18) showed that the addition of 90 mL/L of MO leaves aqueous extract to the drinking water of broilers improved the antioxidant levels, immunity and significantly reduced serum lipid peroxidation levels in broilers. Although MO leaves aqueous extract with antioxidant activity, the composition is complex and the effective components of action cannot be clarified, which has some drawbacks in the in-depth study of specific mechanisms. Therefore, in this study, we extracted and isolated the antioxidant peptides of MO leaves from MLPH and investigated their effects on oxidative damage of HepG2 cells. To clarify the antioxidant components and pre-protective effects of MO leaves on oxidatively damaged HepG2 cells. It provides theoretical support for the study of the antioxidant effect of peptides and provides a basis for the continued study of the antioxidant mechanism of *M. oleifera* leaf peptides.

## Materials and methods

### Materials and chemicals

MO leaves powder was provided by Yunnan Dehong Tianyou Biotechnology Co., Ltd. (Dehong, China). HepG2 hepatocellular carcinoma cells were obtained from Kunming Cell Bank of the Chinese Academy of Sciences (Kunming, China). 1,1-diphenyl-2-picrylhydrazyl (DPPH, 257621), and Dulbecco's Modified Eagle's Medium (DMEM, D0819) were purchased from Sigma (Sigma–Aldrich Chemical Co., St Louis, USA). CAT (BC0200), GSH-Px (BC1190), and MDA (BC0025) kits were purchased from Solarbio Science and Technology Co., Ltd. (Beijing, China), and the SOD (A001-3-2) test kit was obtained from Nanjing Jiancheng Biological Engineering Institute (Nanjing China). Salicylic acid was obtained from Thermo Fisher Scientific Co., Ltd. (Beijing, China), Ascorbic acid (VC, A103535-100g), Hydrogen peroxide solution (H_2_O_2_, H414630-500 mL), ABTS (A276045) was purchased from Aladdin Trading Co., Ltd. (Shanghai, China). Centrifugal filters were purchased from Pall Corporation (Beijing, China). Peptides were synthesized by Anhui Guoping Pharmaceutical Co., Ltd. (Hefei, China). The Reactive Oxygen Assay Kit (BL714A) was purchased from biosharp Biotechnology Ltd. (Beijing, China).

### Preparation of MO leaves proteolytic hydrolysates

Proteins were extracted from MO leaves at 55°C, pH 9.0, a liquid to material ratio of 1:40, and an extraction time of 60 min (Supplementary Figure S3, Table S1) The MLPH were prepared by alkaline protease at an enzymatic digestion time of 5 h, enzyme addition of 3,000 U/g, and enzymatic digestion temperature of 50°C (Supplementary Figures S4, S5, Tables S2, S3). MO leaves proteins fractions were extracted three times simultaneously, and the three fractions obtained were subjected to three experiments. The MLPH membrane ultrafiltration was graded in accordance with the method of Lin et al. (19), after MLPH dialysis, the pH was adjusted to 7. MLPH was sequentially passed through ultrafiltration membranes with MWs of 10, 5, 3, and 1 kDa at a pressure of 0.3 MPa to obtain four different molecular mass fractions, MLPH1 (<1 kDa), MLPH3 (1~3 kDa), MLPH5 (3~5 kDa), and MLPH10 (5~10 kDa), vacuum freeze-dried and stored at −80°C for backup. In this study, the enzymatic digest of MO leaves was sequentially passed through 10, 5, 3, and 1 kDa ultrafiltration membranes in stages that were performed with different retention molecular weights followed by one extract. MLPH1 (< 1 kDa) and MLPH3 (1~3 kDa) were further separated by 1 and 3 kDa centrifugal filters.

### Determination of antioxidant activity

#### Determination of DPPH radical scavenging capacity

The ability of MLPH to scavenge DPPH radicals was investigated using the method of Wang et al. (20) with slight modifications. An ethanol solution of 0.1 mmol/L DPPH was prepared by weighing 4 mg of DPPH dissolved in 100 mL of anhydrous ethanol, placed in a refrigerator at 4°C, and stored in a brown bottle sealed from light. The absorbance values of 1, 3, 5, and 10 kDa samples were measured by taking 0.5 mL of the solution to be measured and 0.5 mL of 0.1 mmol/L ethanol solution in a 2 mL centrifuge tube, protected from light for 50 min, and centrifuged (10 min 4,000 r/min) at a wavelength of 517 nm.


(1) $$\begin{array}{l} \text{DPPH}\;\text{radical}\;\\ \text{scavenging}\;\text{activity}\left(\%\right)=\frac{A_{1}-\left(A_{2}-A_{3}\right)}{A_{1}}\times 100 \end{array}$$

where *A*_1_ is anhydrous ethanol instead of 0.1 mmol/L DPPH; *A*_2_ is 0.5 mL of anhydrous ethanol and 0.5 mL of 0.1 mmol/L DPPH in ethanol solution; and *A*_3_ is 0.5 mL of sample and 0.5 mL of 0.1 mmol/L DPPH in ethanol solution.

#### Determination of hydroxyl radical scavenging capacity

Referring to the method of Xia et al. (21), 8.8 mmol/L H_2_O_2_, 0.5 mL 9 mmol/L Fe^2+^, and 0.5 mL 9 mmol/L salicylic acid-ethanol solutions were added to 100 μL of different concentrations of MLPH. Distilled water was used as the blank Group *A*_0_. The absorbance value *A*_1_ at each concentration was measured at 510 nm, and the hydroxyl radical scavenging activity equation was as follows.


(2) $$\begin{array}{l} \text{Hydroxyl radical scavenging activity(\%)}=\frac{\text{A0-A1}}{\text{A0}}\times 100 \end{array}$$

where *A*_0_ is the light absorption value of the sample group, and *A*_1_ is the light absorption value of the blank group.

#### Superoxide anion radical scavenging capacity

Referring to the method of Miao et al. (22), 2.25 mL of Tris-HCl buffer (0.1 mol/L) with pH 8.2 was added to l mL of distilled water and 1 mL of various concentrations of sample solution, and then 0.25 mL of 10 mmol/L o-triphenol was added. After mixing well, the reaction was heated in a water bath at 25°C, the reaction was aborted by adding 20 μL of HCl at the 3rd min, and the absorbance value was measured at 325 nm. The clearances were calculated as follows.


(3) $$\begin{array}{l} \text{Superoxide anion radical scavenging activity(\%)}\\ \;=\frac{A0-\left(A1-A2\right)}{A0}\times 100 \end{array}$$

where *A*_0_ is the absorbance value of the blank sample with distilled water instead of the sample solution; *A*_1_ is the absorbance value of the sample solution; and *A*_2_ is the absorbance value of the solution without catechol.

#### ABTS radical scavenging capacity

The ABTS radical scavenging activity was referred to the method of Yazdi et al. (23) and Hu et al. (24) with appropriate modifications. First, 2.45 mmol/L potassium persulfate was mixed in a 1:1 ratio with 7 mmol/L ABTS mother liquor, and the reaction was carried out for 12–16 h. Next, the samples were diluted with 5 mM pH 7.4 PBS to achieve an absorbance value of 0.7~0.8 at 734 nm, and finally, the samples were mixed 1:1 with the diluted ABTS free radical working solution, and the reaction was carried out for 10 min at room temperature while protected from light. The calculation formula is as follows.


(4) $$\begin{array}{l} \text{ABTS radical scavenging activity ( c )}=\frac{A0-A1}{A0}\times 100 \end{array}$$

where *A*_0_ is the absorbance value of the blank group, and *A*_1_ is the absorbance value of the sample group.

### MLPH1 for LC-MS/MS analysis

Data acquisition software: Thermo Xcalibur 4.0 (Thermo, USA); reversed-phase column information: C18 column (75 μm × 25 cm, Thermo, USA); chromatographic instrument: EASY—nLC 1,200; mass spectrometer: *Q*—Exactive (Thermo, USA); chromatographic separation time: 90 min A: 2% ACN with 0.1% formic acid; B: 80% ACN with 0.1% formic acid; flow rate: 300 nL/min, EASY—nL C gradient: 0–1 min 5%B, 1–41 min 23%B, 41–51 min 29%B, 51–57 min 38%B, 57–58 min 48% B, 58–59 min 100%B, 59–90 min stop; MS scan range (m/z) 350–1,300, acquisition mode DDA; Top 20 (select the 20 strongest signals in the parent ion for secondary fragmentation); primary mass spectrometry resolution 70,000, fragmentation mode HCD; secondary resolution 17,500, dynamic exclusion. The dynamic exclusion time is 18 s. MS data processing was performed by this software XcaliburTMSoftware3.0 Qual Browser, and the acquired mass spectrometry data were retrieved using the international mainstream proteomics analysis software PEAKS studio 8.5, and the database was downloaded from the Protein Database website using lamu.gene.fasta protein database downloaded from Protein Database website.

### Effect of pretreatment with MO leaves peptides on H_2_O_2_-mediated oxidative damage to HepG2 cells

#### MTT cell viability assay

HepG2 cells in the logarithmic growth phase were inoculated on 96-well plates and adjusted to a concentration of 1 × 10^4^ cells per well. After 24 h of incubation, 200 μL of culture medium was added to the control group. Experimental group cells were treated with different concentrations of LALPVYN (50, 100, 250, 500, 1,000, and 2,000 μg/mL). LALPVYN was added to the blank group without the addition of HepG2 cells. After 24 h of incubation, 5 μg/mL MTT was added to each well for 4 h. The medium was replaced with DMSO, and the OD was measured at a wavelength of 490 nm by a microplate reader. The cell survival rate was calculated by this formula: Survival rate of HepG2 cells (%) = (OD experimental group – OD blank group)/(OD control group – OD blank group) × 100%.

#### Effect of LALPVYN on the viability of HepG2 cells with H_2_O_2_-mediated oxidative damage

After HepG2 cells were cultured for 24 h, 200 μL of the cell suspension was transferred and inoculated into 96-well plates at a density of 1 × 10^4^ cells/well. Then, the cells were divided into a control group, damage group and LALPVYN experimental groups. The control group was prepared by adding 200 μL of culture medium and was then incubated for 24 h. Preparation steps for the damage group: 200 μL DMEM culture solution was added and incubated for 24 h. After incubation, 200 μL H_2_O_2_ (0.5 mmol/L) was added to each well for 4 h. The LALPVYN experimental groups were prepared by adding 200 μL of 50, 100, and 200 μg/mL LALPVYN (dissolved in DMEM) and were then incubated for 24 h. The positive control was prepared by adding 60 μg/mL of Vc and were then incubated for 24 h. The blank group was prepared by adding LALPVYN without HepG2 cells and then incubated for 24 h. The MTT method was used to measure cell viability.

#### Analysis of intracellular antioxidant capacity of HepG2

HepG2 cells (3 × 10^5^) were pipetted separately in 6 (60 mm) cell culture dishes and cultured for 24 h. After performing different treatments (the experimental treatment is the same as 2.5.2), total superoxide dismutase (SOD) and catalase (CAT) and Glutathione peroxidase (GSH-Px) activities and malondialdehyde (MDA) contents in cell lysis products were determined according to the method in the SOD, CAT, GSH-Px, and MDA kit instructions.

The Reactive Oxygen Assay Kit is a kit for the detection of reactive oxygen species using the fluorescent probe H2DCFDA.H2DCFDA itself is non-fluorescent and can freely pass through the cell membrane. Once inside the cell, it can be hydrolysed by intracellular esterases to generate DCFH, which is not permeable to the cell membrane, thus allowing the probe to be easily loaded into the cell. The level of intracellular reactive oxygen species can be determined by measuring the fluorescence of DCFH, which oxidizes non-fluorescent DCFH to produce fluorescent DCF. Based on the production of fluorescence in living cells, the level and variation of cellular reactive oxygen species can be determined.

ROS was measured using the Reactive Oxygen Species Assay Kit, HepG2 cells were inoculated with 2 × 10^5^ cells/well in laser confocal dishes in combination with the method of Ladda et al. (25), and the cells were treated according to the experimental grouping method in 2.5.2. Pictures were taken with a laser confocal microscope, and fluorescence intensity analysis was performed by *ImageJ*.

#### Apoptosis assay

HepG2 cells were inoculated on 6-well plates at 3 × 10^5^ cells/well, and the apoptosis rate of HepG2 cells was detected by flow cytometry (Ex = 488 nm, FL1 Em = 525 ± 20 nm, FL2 Em = 585 ± 21 nm) and BD Accuri C6 software according to the instructions of the Annexin V-FITC kit.

### Statistical analysis

All experiments were performed with three independent experiments. The results are expressed as the mean ± SD. SPSS version 19.0 (SPSS Institute, Chicago, USA) and one-way ANOVA were applied to analyze the significance of the results. The significant differences were set at *p* < 0.05 achieved by Duncan's multiple range Test.

## Results and discussion

### *In vitro* antioxidant activity of MLPH

A free radical is an atomic or ionic group with unpaired electrons formed by a compound molecule due to the breakage of a covalent bond. Free radicals usually have the tendency to gain or lose electrons, so they are chemically very active and can easily react with other substances in the body to form new free radicals or oxides. When the body's free radical homeostasis is disrupted, too many free radicals accumulate. When this happens, free radicals, as cytotoxic oxidants, can cause damage to proteins, nucleic acids, and lipids in the body, which can lead to the destruction of cell structure and even to the mutation of cells, posing a serious risk.

**Figure 1:**
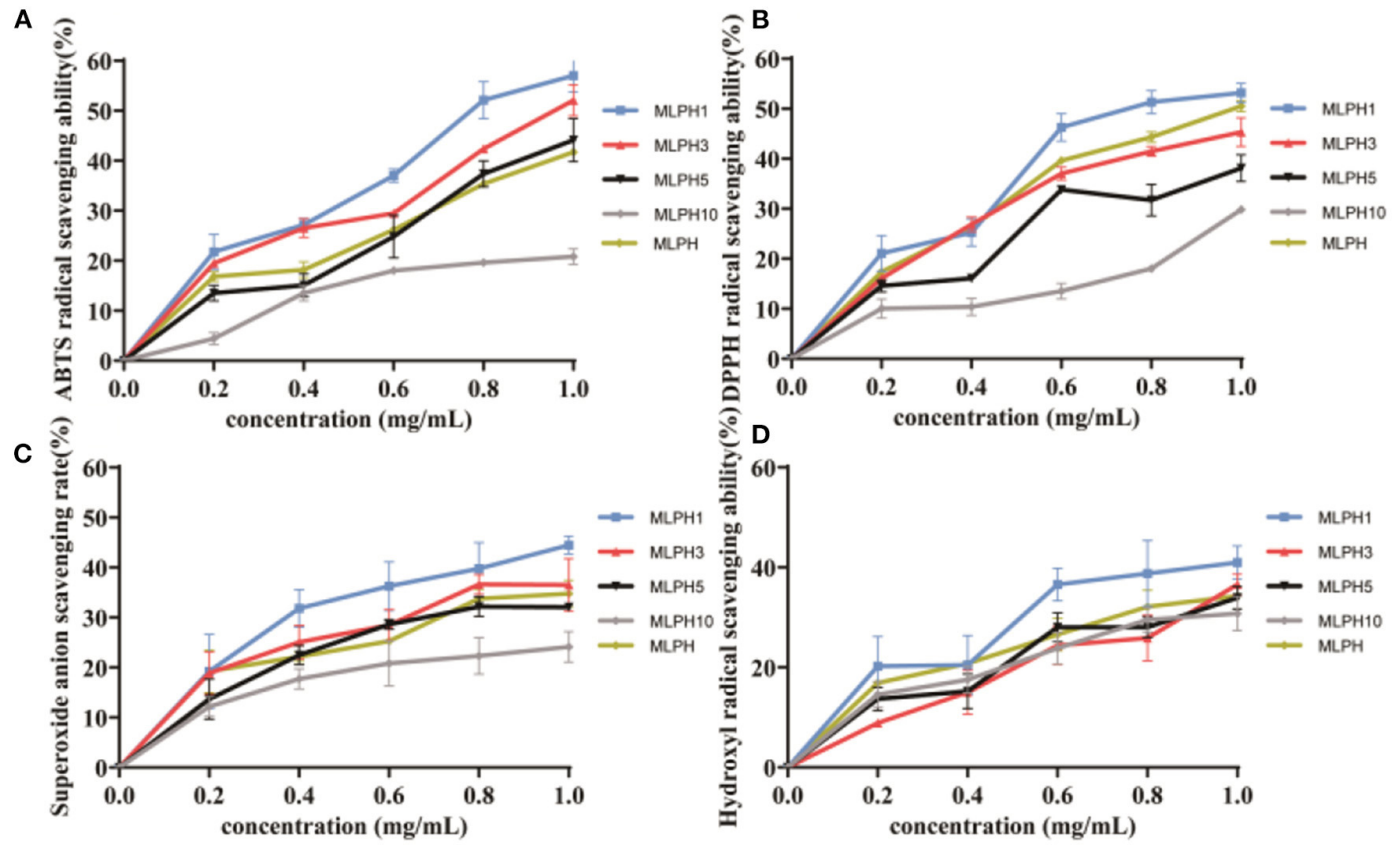
Antioxidant activity of MLPH. **(A)** ABTS free radical scavenging ability; **(B)** DPPH free radical scavenging ability; **(C)** superoxide anion radical scavenging ability; **(D)** hydroxyl radical scavenging ability; MLPH1 is <1 kDa *M. oleifera* leaves proteolytic, MLPH2 is 1~3 kDa *M. oleifera* leaves hydrolysates, MLPH5 is 3~5 kDa *M. oleifera* leaves proteolytic, MLPH10 is 5~10 kDa *M. oleifera* leaves proteolytic. Data are expressed as mean ± SD from three independent experiments (*n* = 3).

The scavenging rates of ABTS, DPPH, superoxide anion, and hydroxyl radical by MLPH are shown in Figure 1. MLPH of different molecular weights all showed scavenging activity against free radicals. Among them, MLPH1 has a stronger ability to scavenge free radicals and MLPH10 has a weaker ability to scavenge free radicals.This is consistent with the results of Graziani et al. (26) who studied small-molecular-weight antioxidant peptides from kidney beans, and Luo et al. (27) who studied small-molecular-weight antioxidant peptides from buckwheat with strong scavenging activity. It has been shown that the small molecular weight fractions are all more active in scavenging free radicals because they facilitate binding to the target molecules (28). The ABTS scavenging activity scavenging IC_50_ value of the MLPH1 fraction was 0.83 mg/mL, the DPPH scavenging IC_50_ value was 0.82 mg/mL, the superoxide anion scavenging IC_50_ value was 1.35 mg/mL and the hydroxyl radical scavenging IC_50_ value was 1.58 mg/mL, which shows a trend of enhancement with increasing concentration. This is consistent with the results of the Zhike et al. (29) study, in which collagen peptides with different molecular weights of bovine collagen had stronger scavenging activity with increasing concentrations. In summary, MLPH with a smaller molecular weight has stronger antioxidant activity, and the scavenging effect of MLPH3 on free radicals is similar to that of MLPH1. MLPH1 and MLPH3 were studied next.

### LC-MS/MS identification results

#### Amino acid composition

The antioxidant capacity of peptides is closely related to the type and number of amino acids and amino acid arrangement of the constituent peptides. Many amino acids and their derivatives have the ability to scavenge free radicals, such as lysine (K), histidine (H), tyrosine (Y), methionine (M), proline (P), argnine (R), glutamine (E), and cysteine (C) (30). To further clarify the strong antioxidant activity of the small molecule MO leaves proteolytes, MLPH1 and MLPH3 were separated by centrifugal filters to obtain fractions MLPH1-1 and MLPH3-1, and the amino acid species and mass fractions are shown in Table 1.

**Table 1:** Amino acid composition of different molecular weight MO leaves proteolytic hydrolysates.

**Amino acid name**	**Quality score/%**		**Amino acid name**	**Quality score/%**	
**1 kDa**	**MLPH1-1**	**MLPH1**	**3 kDa**	**MLPH3-1**	**MLPH3**
Glycine (G)	3.81	3.03	Glycine (G)	3.89	3.1
Alanine (A)	5.42	6.12	Alanine (A)	5.25	4.5
Valine (V)	4.66	8.05	Valine (V)	6.36	9.14
Leucine (L)	12.05	14.31	Leucine (L)	12.7	15.06
Isoleucine (I)	7.49	9.01	Isoleucine (I)	7.54	5.42
Phenylalanine (F)	3.56	3.34	Phenylalanine (F)	5.46	2.28
Tryptophan (W)	1.61	0.3	Tryptophan (W)	1.41	0.94
Tyrosine (Y)	3.05	4.39	Tyrosine (Y)	4.04	3.33
Aspartic acid (D)	6.06	2.69	Aspartic acid (D)	6.62	2.44
Asparagine (N)	2.68	9.08	Asparagine (N)	3.18	9.1
Glutamate (E)	9.64	1.78	Glutamate (E)	9.28	3.38
Lysine (K)	7.5	14.77	Lysine (K)	7.44	6.04
Glutamine (Q)	5.54	2.96	Glutamine (Q)	3.4	2.68
Methionine (M)	1.41	1.21	Methionine (M)	1.32	2.06
Serine (S)	5.78	5.1	Serine (S)	4.45	7.24
Threonine (T)	3.98	5.3	Threonine (T)	4.53	4.92
Cysteine (C)	0.73	0.70	Cysteine (C)	0.29	0.56
Proline (P)	3.67	5.58	Proline (P)	3.46	6.34
Histidine (H)	2	1.88	Histidine (H)	1.1	4.27
Arginine (R)	9.35	1.41	Arginine (R)	8.28	7.2
Total	99.99	100	Total	100

Mass fraction of amino acids = (molecular weight of amino acids ^*^ number of amino acids)/total mass of amino acids.

Table 1 shows that MLPH1 and MLPH3 showed increased levels of aspartate (D), glutamate (E), glutamine (Q), serine (S), and arginine (R) after centrifugation and filtration. The scavenging activity of MLPH1, MLPH1-1, MLPH3, and MLPH3-1 against ABTS radicals was also verified and the results are shown in Supplementary Figure S1. The results shown in Supplementary Figure S1 indicate that MLPH1-1 showed better scavenging activity.

#### Molecular weight and sequence identification of MLPH1-1

The results of characteristics of peptides from MLPH1-1 are shown in Table 2. MLPH1-1 consists of a mixture of 20 peptides with molecular weights between 722.4329~1,791.7952 Da, consisting of 6~16 amino acids; more than 50% of them contain proline (P), glycine (G), alanine (A), valine (V), and leucine (L) with potential antioxidant activity (2). The peptide chain contains aromatic rings, imidazole groups, and sulfur-containing groups that can enhance the antioxidant activity of the peptides.

**Table 2:** Characteristics of peptides from MLPH1-1 and identified by LC-MS/MS.

	**Amino acid sequence**	**Molecular weight (Da)**	**Peak area**
1	TVLIMELINNVAK	1,456.8323	5.69 × 10^5^
2	QIKTIPKKPN	1,165.7183	1.87 × 10^7^
3	GAVGSGLSK	774.4235	3.37 × 10^4^
4	LIKVLLTAVKDF	1,358.8536	5.56 × 10^6^
5	KAPAYSV	734.3962	9.38 × 10^5^
6	TFLKSK	722.4326	4.19 × 10^5^
7	LALPVYN	788.4432	2.83 × 10^5^
8	EYDLSKAQ	952.4501	3.51 × 10^4^
9	FHEEDDAKLF	1,249.5615	1.09 × 10^8^
10	LDEGKWQHVK	1,238.6407	1.01 × 0^6^
11	PKGLKTN	756.4493	4.01 × 10^5^
12	LHIAALVFQ	1,010.5912	1.20 × 10^5^
13	KAYGQNLSIGYDDFDS	1,791.7952	1.84 × 10^5^
14	GHVALVFVN	954.5287	2.41 × 10^6^
15	LLVVSGIN	813.496	1.08 × 10^6^
16	TVNIISSKR	1,016.5978	9.55 × 10^6^
17	SKSLIAIN	844.5018	2.18 × 10^6^
18	HTELALKYVN	1,186.6346	8.65 × 10^4^
19	GPIILPN	722.4326	2.69 × 10^7^
20	PAALAKMKN	942.532	4.30 × 10^5^

**Figure 2:**
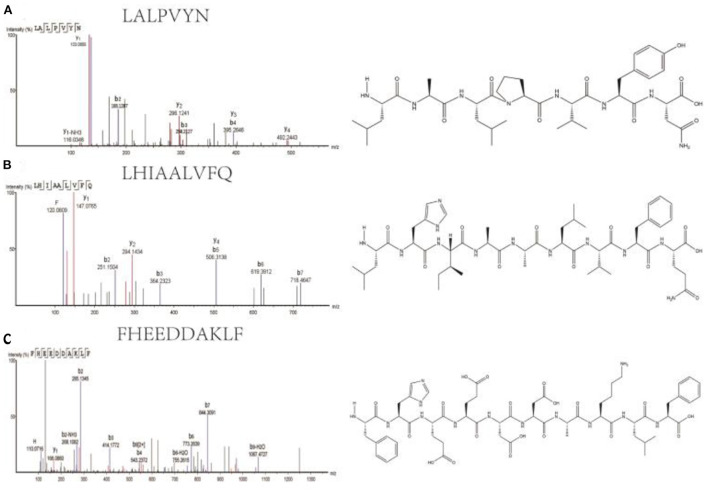
Secondary mass spectra and chemical structures of LALPVYN **(A)**, LHIAALVFQ **(B)**, and FHEEDDAKLF **(C)**.

A total of three peptides with potentially high antioxidant activity were selected based on the following conditions. (1) short peptides with a number of amino acids of 2–10 (31, 32); (2) aromatic amino acid residues, such as phenylalanine (F), tryptophan (W), and tyrosine (Y) (33); (3) two specific residues, proline and valine residues (P and V), leucine and alanine residues (L and A), or both proline and leucine residues (P and L) (34); and (4) one or more hydrophobic amino acid residues (35–37). The three peptides selected were LALPVYN, LHIAALVFQ, and FHEEDDAKLF. The secondary mass spectra of the three peptides are shown in Figure 2.

### Determination of antioxidant capacity of synthetic peptides

**Figure 3:**
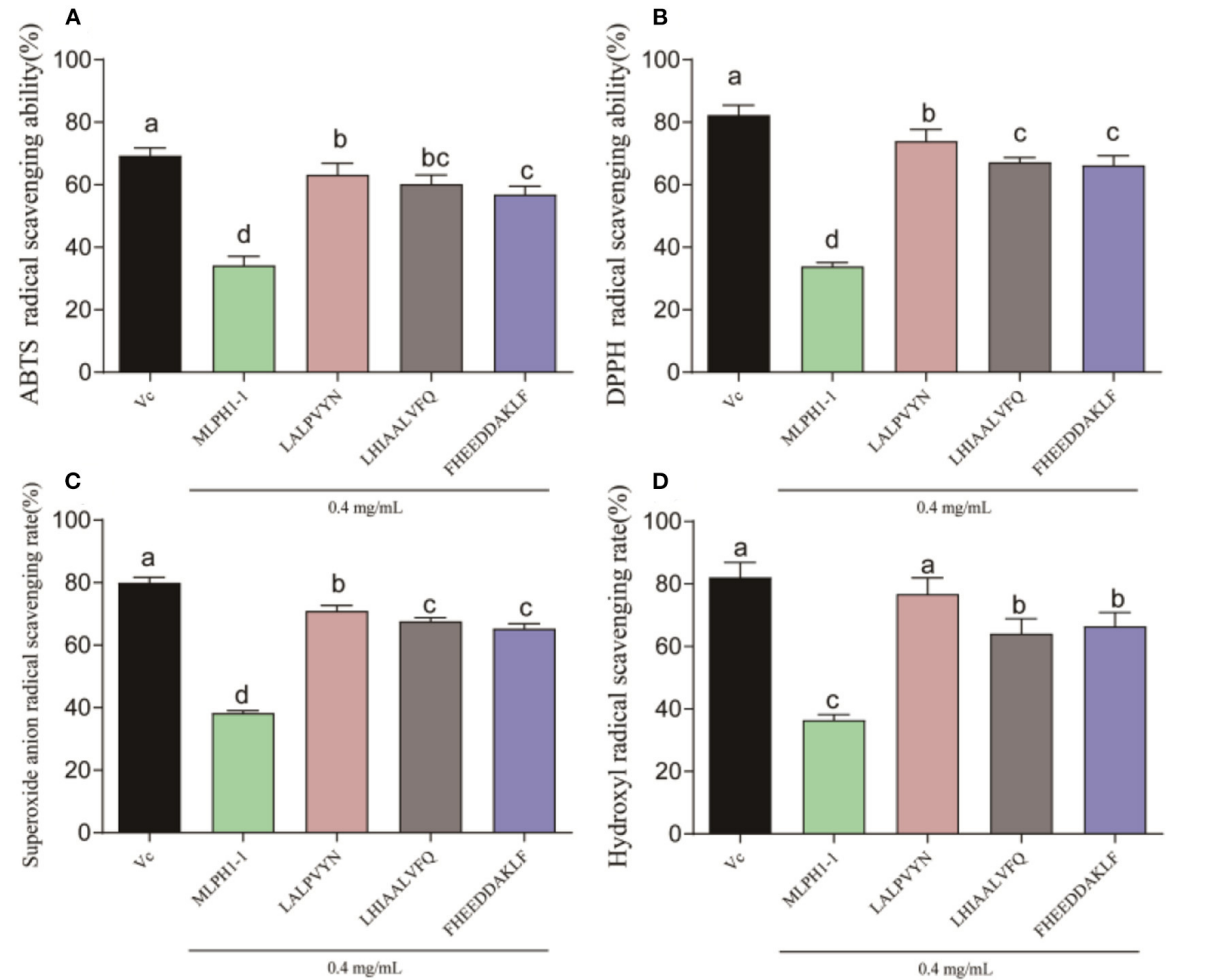
Determination of the antioxidant capacity of each peptide. **(A)** ABTS radical scavenging ability; **(B)** DPPH radical scavenging ability; **(C)** superoxide anion radical scavenging ability; **(D)** hydroxyl radical scavenging ability. Data are expressed as mean ± SD from three independent experiments (*n* = 3), results marked with the same letters were not significantly different (*P* > 0.05).

As shown in Figure 3, all three synthetic peptides (LALPVYN, LHIAALVFQ, and FHEEDDAKLF) showed stronger antioxidant activity, which was significantly higher than that of the MLPH1-1. The ABTS, DPPH, superoxide anion, and hydroxyl radical scavenging rates of LALPVYN at 0.4 mg/mL were 63.58 ± 1.75, 72.65 ± 2.01, 70.93 ± 1.02, and 75.42 ± 4.08%, respectively, with higher antioxidant effects than other amino acid sequences and close to VC. Meanwhile, the antioxidant effect of LALPVYN is superior to that of the RRPB3III fraction isolated and purified from rice pomace protein (38), exhibiting better antioxidant activity.

### Effects of LALPVYN pretreatment on the survival rate of H_2_O_2_-mediated oxidative injured HepG2 cells

Human hepatocellular carcinoma cells (HepG2) are easy to culture and highly representative. HepG2 cells are present in the liver and the liver plays an important role in the antioxidant process of the body (39). Therefore, the oxidative damage model of HepG2 cells by chemical agents is often chosen to evaluate the protective effects of natural antioxidants and phytochemicals on the liver.

H_2_O_2_ is a well-known hepatotoxic chemical with a long half-life that can be directly converted into hydroxyl radicals and oxygen radicals, which are important triggers of oxidative stress damage in the body (40). In order to show the antioxidant activity of LALPVYN, a model of oxidative damage in HepG2 cells was established using H_2_O_2_ to assess its antioxidant activity.

**Figure 4:**
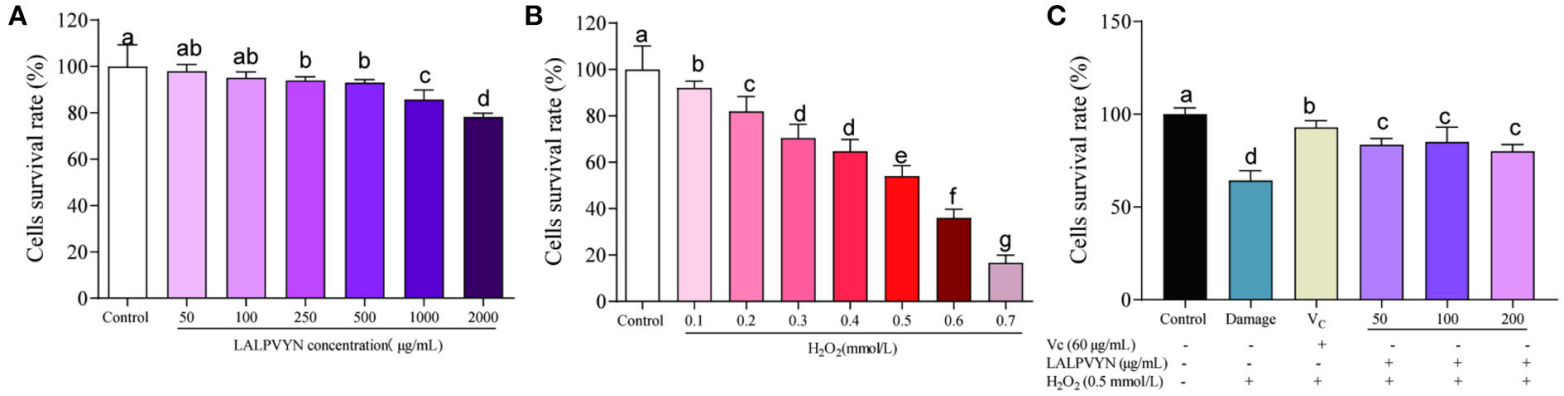
Effects of LALPVYN pretreatment on the survival rate of H_2_O_2_-mediated oxidative injured HepG2 cells. **(A)** The survival rates of HepG2 cells treated with LAL at different concentration; **(B)** the survival rates of HepG2 cells treated with H_2_O_2_ at different concentration; **(C)** the survival rates of HepG2 cells treated with both LALPVYN, V_C_ and H_2_O_2_. Data are expressed as mean ± SD from three independent experiments (*n* = 3), results marked with the same letters were not significantly different (*P* > 0.05).

The antioxidant effect of LALPVYN is shown in Figure 4. First, the effect of LALPVYN on the survival rate of HepG2 cells was investigated (Figure 4A). The results showed that LALPVYN had no growth-promoting effect on HepG2 cells, and the survival rate of HepG2 cells showed a downward trend with increasing concentration. Cell survival in the range of 50–500 μg/mL was 97.95 ± 1.18–93.03 ± 0.56%, with no toxic effect on cells (>90%) compared to the control group. When the concentration of LALPVYN reached 1,000 μg/mL, it was significantly reduced compared with the control group and had toxic effects on cells. Concentrations of 50, 100, and 200 μg/mL were selected for the experiment under comprehensive consideration.

Second, oxidative damage was modeled (Figure 4B). The results showed that the cells were induced with different concentrations of H_2_O_2_ for 4 h, and the cell survival rate decreased with increasing concentration. The survival rate of cells induced by 0.5 mmol/L H_2_O_2_ for 4 h was 54.09 ± 1.58%, which was close to the concentration of semi-inhibited cells, and the concentration of the established cellular oxidative damage model was determined.

Finally, the antioxidant effect of LALPVYN was investigated (Figure 4C). The results showed that the cell survival rate after LALPVYN pretreatment ranged from 80.12 ± 1.47 to 85.11 ± 3.24%, which was close to the cell survival rate of VC pretreatment, indicating that pretreatment with MO leaves antioxidant peptides could effectively reduce the oxidative damage effect on cells.

### Effect of LALPVYN pretreatment on SOD, CAT, GSH-Px, and MDA of H_2_O_2_-mediated oxidative injured HepG2 cells

The antioxidant capacity of intracellular antioxidants can be categorized into direct and indirect aspects. The direct antioxidant properties of antioxidants are achieved by providing hydrogen atoms or electrons to remove intracellular ROS. The indirect antioxidant properties of antioxidants are mediated by the expression of antioxidant enzymes and antioxidant genes to resist cellular oxidative damage (41). It has been demonstrated that the intracellular antioxidant enzyme system plays an important role in protecting against oxidative stress damage. To elucidate the mechanism of the protective effect of LALPVYN on H_2_O_2_-mediated oxidative stress injury in HepG2 cells, the effects of LALPVYN on the CAT, GSH-Px, SOD, and MDA contents of HepG2 cells were investigated.

**Figure 5:**
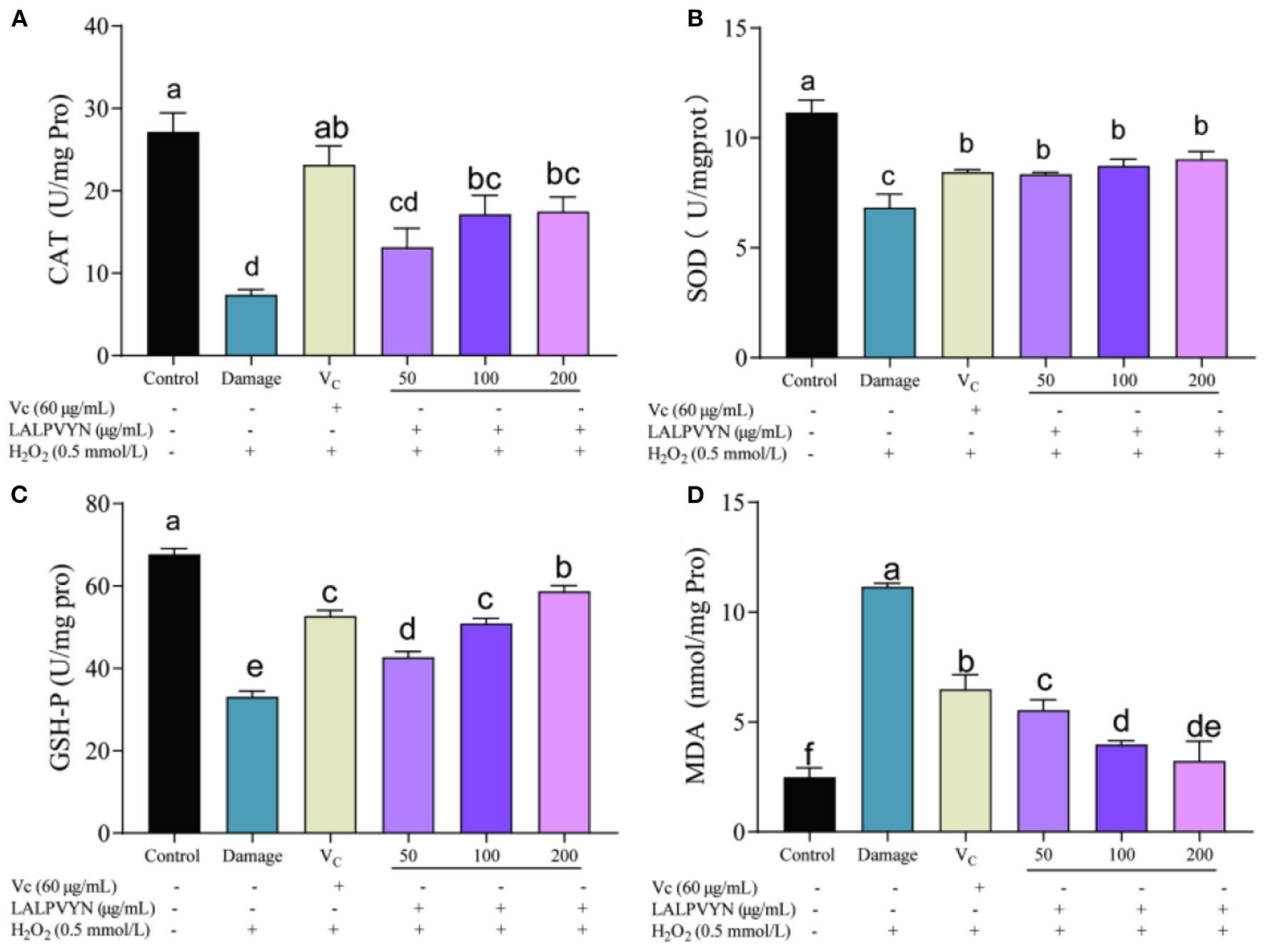
Effect of LALPVYN pretreatment on SOD, CAT, GSH-Px, and MDA of H_2_O_2_-mediated oxidative injured HepG2 cells. **(A)** Effect of LALPVYN on CAT, **(B)** effect of LALPVYN on GSH-Px; **(C)** effect of LALPVYN on SOD; **(D)** effect of LALPVYN on MDA. Data are expressed as mean ± SD from three independent experiments (*n* = 3), results marked with the same letters were not significantly different (*P* > 0.05).

An important biomarker of the cellular response to oxidative stress is the change in antioxidant enzyme activity. The usual mitigation of oxidative stress is *via* the conversion of highly reactive superoxide anions to hydrogen peroxide (H_2_O_2_), catalyzed by superoxide dismutase (SOD). H_2_O_2_ is then broken down by catalase (CAT) into water and oxygen. As shown in Figure 5A, the CAT enzyme activity was higher in untreated cells (27.15 ± 2.29 U/mg pro) and lowest in the damage group (7.38 ± 0.63 U/mg pro), and the pre-protected CAT enzyme activities by LALPVYN were all increased to different degrees compared to those of the damage group, which were 13.15 ± 2.29, 17.15 ± 2.13, and 17.50 ± 1.75 U/mg pro. From Figure 5B, the SOD enzyme activity was 11.16 ± 0.32 U/mg Pro in untreated cells, the lowest enzyme activity was 6.83 ± 0.35 U/mg Pro in the damaged group, and after pretreatment with LALPVYN, the SOD enzyme activity was 8.33 ± 0.053, 8.74 ± 0.17, and 9.04 ± 0.19 U/mg Pro, which were significantly increased.

Peroxidase (GSH-Px) is widely present in the organism and its role is to catalyze the change of GSH to GSSG, which reduces toxic peroxides to non-toxic hydroxyl compounds. It also promotes the decomposition of H_2_O_2_ and protects the structure and function of cell membranes from interference and damage by peroxides. From Figure 5C, compared to the control group (67.72 ± 1.36 Umg/Pro), the GSH-Px enzyme activity within the damage group was significantly reduced to 33.06 ± 1.46 Umg/Pro. The group preprotected by LALPVYN showed an increase in GSH-Px enzyme activity compared to the damage group, and the GSH-Px enzyme activity after treatment with LALPVYN was 42.72 ± 1.36, 50.93 ± 1.25, and 58.72 ± 1.36 Umg/Pro.

Malondialdehyde (MDA) is a three-carbon compound that is an indicator of lipid peroxidation and is a component of the breakdown of peroxidized polyunsaturated fatty acids (42). ROS produced by cells during metabolism can be associated with phospholipids, enzymes, and membrane receptors of biological membranes with polyunsaturated fatty acids, triggering lipid peroxidation to form MDA. MDA is an important indicator of oxidative cellular damage, and its higher level indicates more severe oxidative cellular damage. From Figure 5D, the MDA level in the control group of cells was low at 2.49 ± 0.25 nmol/mg pro, and the MDA level in the damaged group was significantly higher at 11.16 ± 0.088 nmol/mg pro as compared to the control group. Compared with the model group, the MDA content was significantly lower in the group preprotected by LALPVYN (50, 100, and 200 μg/mL), with MDA contents of 3.49 ± 0.28, 5.54 ± 0.11, and 6.97 ± 0.52 nmol/mg pro, respectively, and showed a significant dose-effect relationship with the LALPVYN. This indicates that MO leaves antioxidant peptides can effectively inhibit lipid peroxidation and reduce the production of malondialdehyde. The reason may be that LALPVYN contains hydrophobic amino acids, which have high binding power to hydrophobic polyunsaturated fatty acids and have some protective effect on cellular oxidative damage (43).

In summary, LALPVYN not only scavenges intracellular free radicals, but also enhances endogenous antioxidant defense systems, including antioxidant enzyme defense systems and glutathione systems, thereby reducing H_2_O_2_-induced MDA and free radical production in HepG2 cells, and achieving cell protection. This is consistent with the results of Wen et al. (44) study which showed that the antioxidant peptide treatment of watermelon seeds significantly attenuated the H_2_O_2_-induced decrease in antioxidant enzyme activity. The antioxidant effect was superior to that of the duck embryonic peptide studied by He et al. (45), and a smaller dose of LALPVYN significantly alleviated the effect of reduced antioxidant enzyme activity.

### Effect of LALPVYN pretreatment on ROS of H_2_O_2_-mediated oxidative injured HepG2 cells

Studies have shown that excess ROS causes the oxidation of proteins and lipids, which in turn leads to the disruption of nuclear DNA and mitochondrial integrity and ultimately, to cell death (39, 46). DCFH-DA is a specific fluorescent probe for intracellular reactive oxygen species that penetrates the cell membrane and is degraded by membrane esterases to DCFH, which further binds specifically to intracellular ROS to generate DCFH that can emit fluorescent signals.

**Figure 6:**
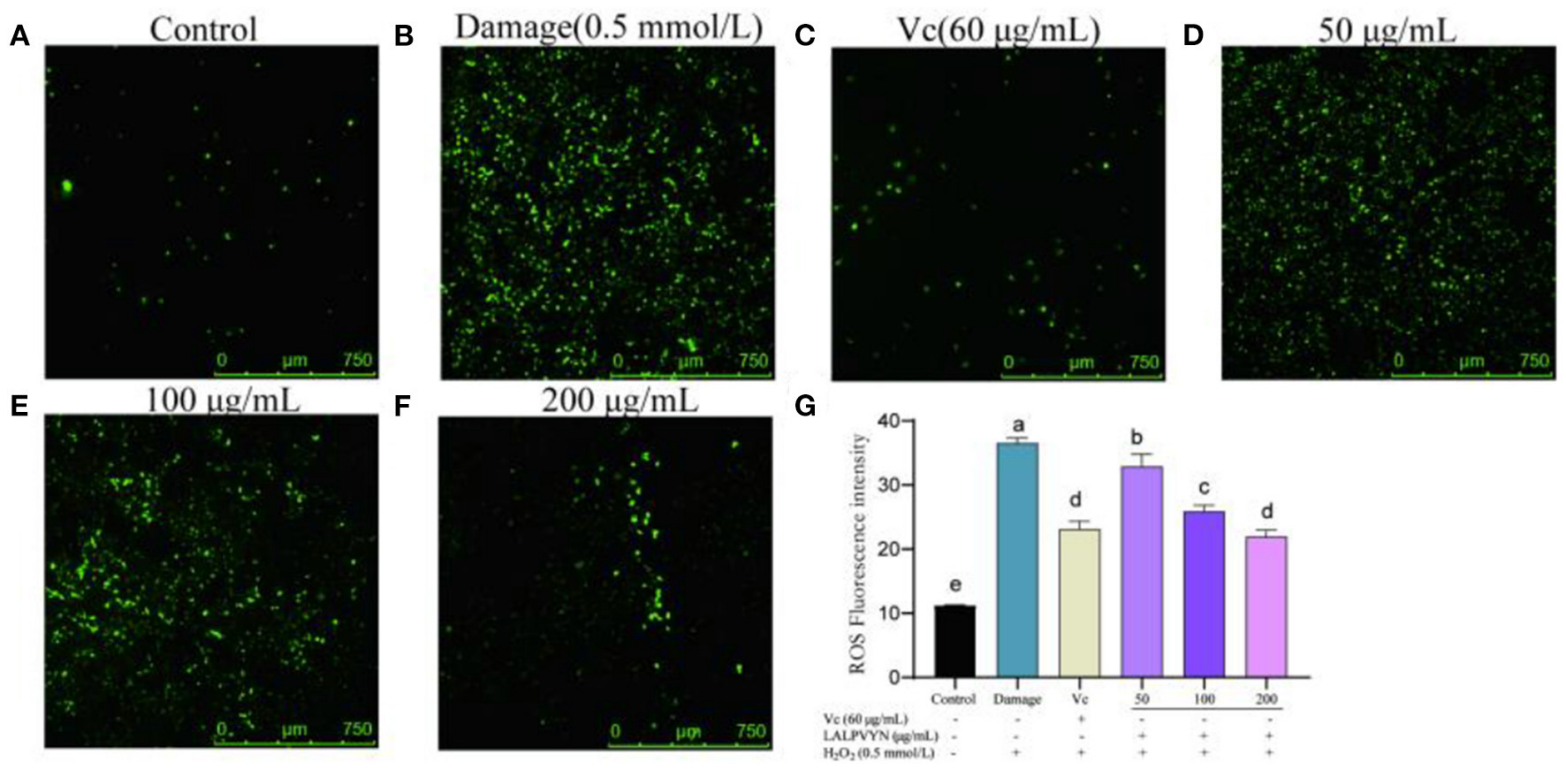
The effect of LALPVYN pretreatment on ROS of H_2_O_2_-mediated oxidative injured HepG2 cells. **(A–F)** Pictures were taken by laser scanning confocal microscopy; **(G)** the average fluorescence intensity was analyzed by Image J. Data are expressed as mean ± SD from three independent experiments (*n* = 3), results marked with the same letters were not significantly different (*P* > 0.05).

The effect of LALPVYN on H_2_O_2_-mediated ROS content in HepG2 cells is shown in Figure 6. The mean fluorescence intensity of ROS in the damaged group (36.57 ± 0.44) was significantly different compared to the control group (11.26 ± 0.027), indicating that H_2_O_2_ can cause an increase in the level of ROS in HepG2 cells. The mean fluorescence intensity of ROS was significantly lower in the group preprotected by LALPVYN than in the H_2_O_2_ damage group (32.93 ± 1.1, 25.92 ± 0.51, and 24.99 ± 0.57), where the ROS content of the fraction with a peptide concentration of 200 μg/mL was close to that of the positive control VC group (23.14 ± 0.69). This cytoprotective effect of LALPVYN may be due to its antioxidant ability to scavenge intracellular ROS. Similar results were reported by Yi et al. (47), who reported that soy peptides could exhibit the ability to scavenge intracellular ROS. The above results suggest that LALPVYN can effectively protect HepG2 cells from free radical damage.

### Effect of LALPVYN pretreatment on apoptosis of H_2_O_2_-mediated oxidative injured HepG2 cells

Normal apoptosis, also known as programmed cell death, is an active, orderly process of cell death determined by multiple genes. Abnormal apoptosis is associated with many human diseases and external influences, such as ischemic injury, autoimmune diseases and cancer. Apoptosis analysis is always used to evaluate the potential use of bioactive ingredients as food supplements or chemotherapeutic agents (48).

**Figure 7:**
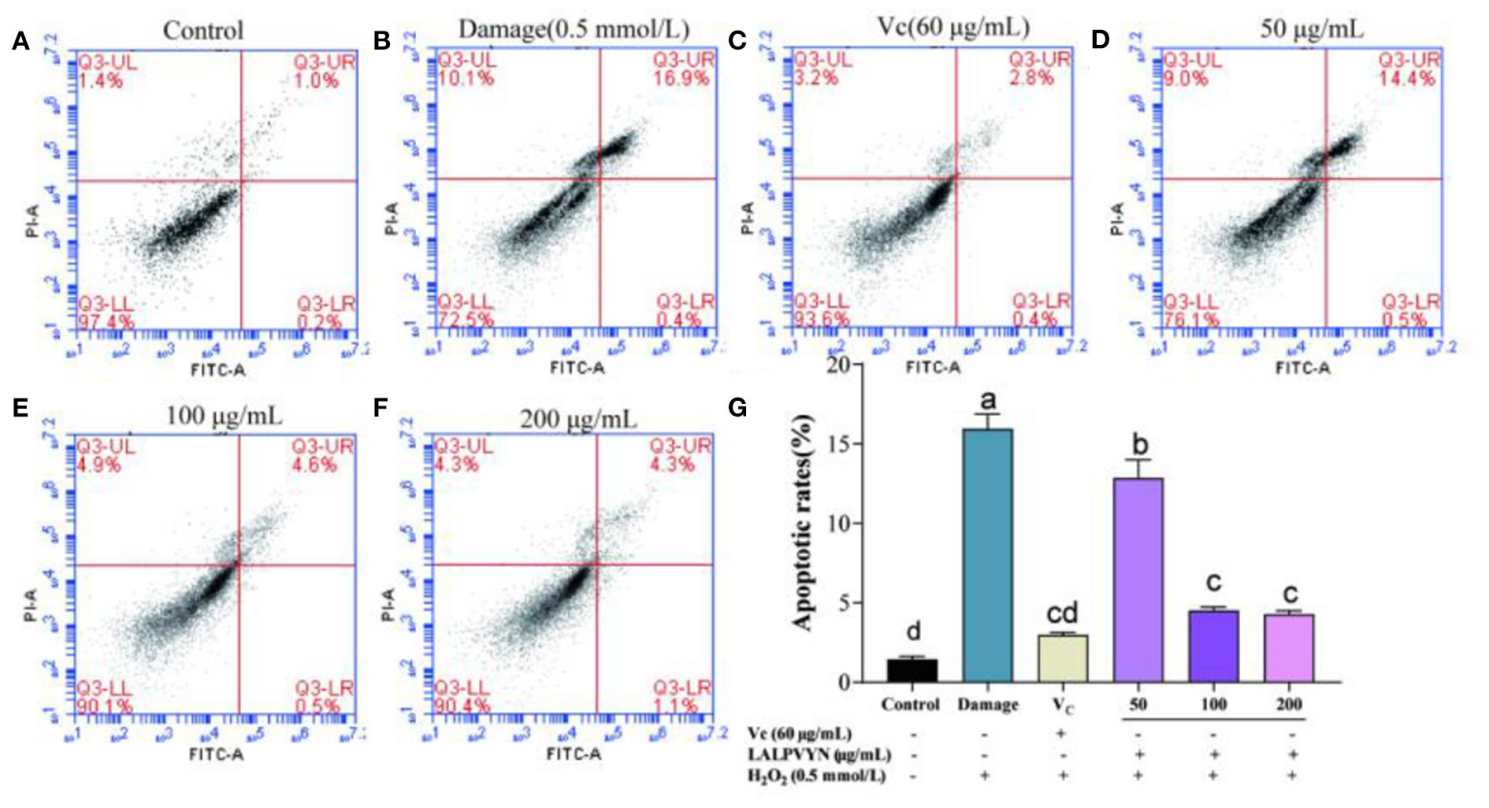
The effect of LALPVYN pretreatment on apoptosis of H_2_O_2_-mediated oxidative injured HepG2 cells. **(A–F)** Cell cycle was analyzed by flow cytometry; **(G)** analysis of apoptotic rate by flowjo software. Data are expressed as mean ± SD from three independent experiments (*n* = 3), results marked with the same letters were not significantly different (*P* > 0.05).

From Figure 7, HepG2 cells were treated with different concentrations of LALPVYN (50, 100, and 200 μg/mL), and the effect of LALPVYN on the apoptosis of HepG2 cells was detected by flow cytometry. The apoptosis rate of HepG2 cells in the control group was low at 1.43 ± 0.19%, while the apoptosis rate in the damage group was significantly increased at 15.93 ± 0.93%. The apoptosis rate decreased significantly when cells were treated with 50–200 μg/mL LALPVYN, with the lowest apoptosis rate of 4.28 ± 0.22% for 200 μg/mL LALPVYN. Consistent with the results of Hu et al. (49) study, the apoptosis rate in the 200 μg/mL LALPVYN-treated group was lower than that in the 0.19 mg/mL GSGH-treated group. This finding indicates that LALPVYN is more effective than GSGH in alleviating apoptosis induced by oxidative stress. In summary, the results indicate that LALPVYN has a protective effect on H_2_O_2_-mediated oxidative stress injury, with the greatest protective effect of LALPVYN occurring at 200 μg/mL.

## Conclusions

The preparation and antioxidant activity of peptides from MO leaves have rarely been reported. In this study, MO leaves protein hydrolysate was prepared using alkaline protease and separated by membrane filtration and centrifugal separator. Three peptides with high antioxidant activity were obtained from MO leaves protein hydrolysate and the structures of the three antioxidant peptides were clarified. The LALPVYN peptide with a molecular weight of 788.44 Da outperformed other peptides in terms of free radical scavenging. It has a good protective effect on H_2_O_2_-induced oxidative damage in HepG2 cells, effectively increasing CAT, GSH-PX, and SOD activities, reducing MDA and ROS content, and inhibiting apoptosis. The antioxidant activity of LALPVYN has some potential compared to VC and MLPH has the potential to become a natural antioxidant in food. Although LALPVYN has strong antioxidant activity *in vitro*, whether it has a strong antioxidant effect *in vivo* needs to be further explored. We do not know whether the peptides can pass through the complex gastrointestinal environment of the body and thus exert their antioxidant effect. In future work, further studies on LALPVYN are still needed, with the aim of clarifying whether LALPVYN also possesses the same antioxidant activity in mice as in previous studies. Further insights into the antioxidant mechanism of LALPVYN are needed.

## Data availability statement

The original contributions presented in the study are included in the article/Supplementary material, further inquiries can be directed to the corresponding authors.

## Author contributions

JS and YT supervised the study. YT, LT, and FG designed experiments. LT and FG contributed equally to this work. FG, YL, and X-ZW performed experiments. FG wrote the manuscript. YT, LT, and MY reviewed the manuscript and results. All authors contributed to the article and approved the submitted version.

## Funding

This work was supported by Major Project of Science and Technology Department of Yunnan Province (202002AA100005 and 202102AE090027-2), Cassava Industrial Technology System of China (CARS-11-YNTY), Yunnan Province Ten Thousand Plan Industrial Technology Talents project (YNWR-CYJS-2020-010), and R&D and application of key technologies for specialty new functional and nutritional health foods (2019ZG00905).

## Conflict of interest

The authors declare that the research was conducted in the absence of any commercial or financial relationships that could be construed as a potential conflict of interest.

## Publisher's note

All claims expressed in this article are solely those of the authors and do not necessarily represent those of their affiliated organizations, or those of the publisher, the editors and the reviewers. Any product that may be evaluated in this article, or claim that may be made by its manufacturer, is not guaranteed or endorsed by the publisher.
