# Supplementary material for: Preparation of antioxidant peptides from Moringa oleifera leaves and their protection against oxidative damage in HepG2 cells
Source: Front Nutr. 2022 Dec 1;9:1062671. doi: 10.3389/fnut.2022.1062671 (PMC9751868; doi:10.3389/fnut.2022.1062671)
Supplement: Supplementary file 1 [file Data_Sheet_1.docx]

Supplementary Material

Figure S1 Scavenging ability of different fractions on ABTS free radicals


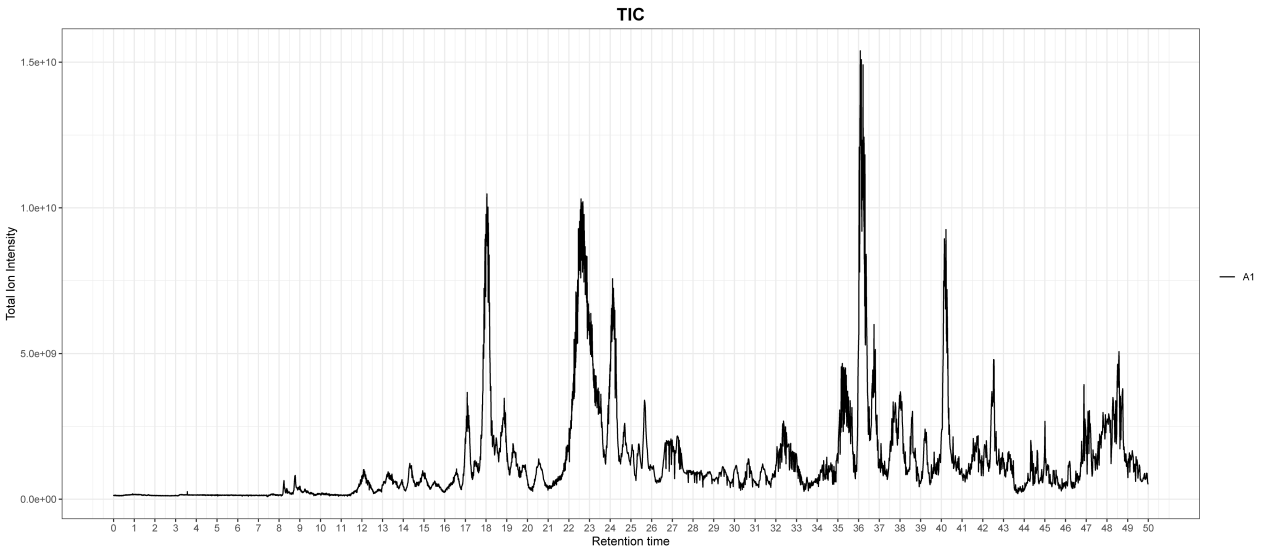


Figure S2 LC chromatogram of MLPH1-1


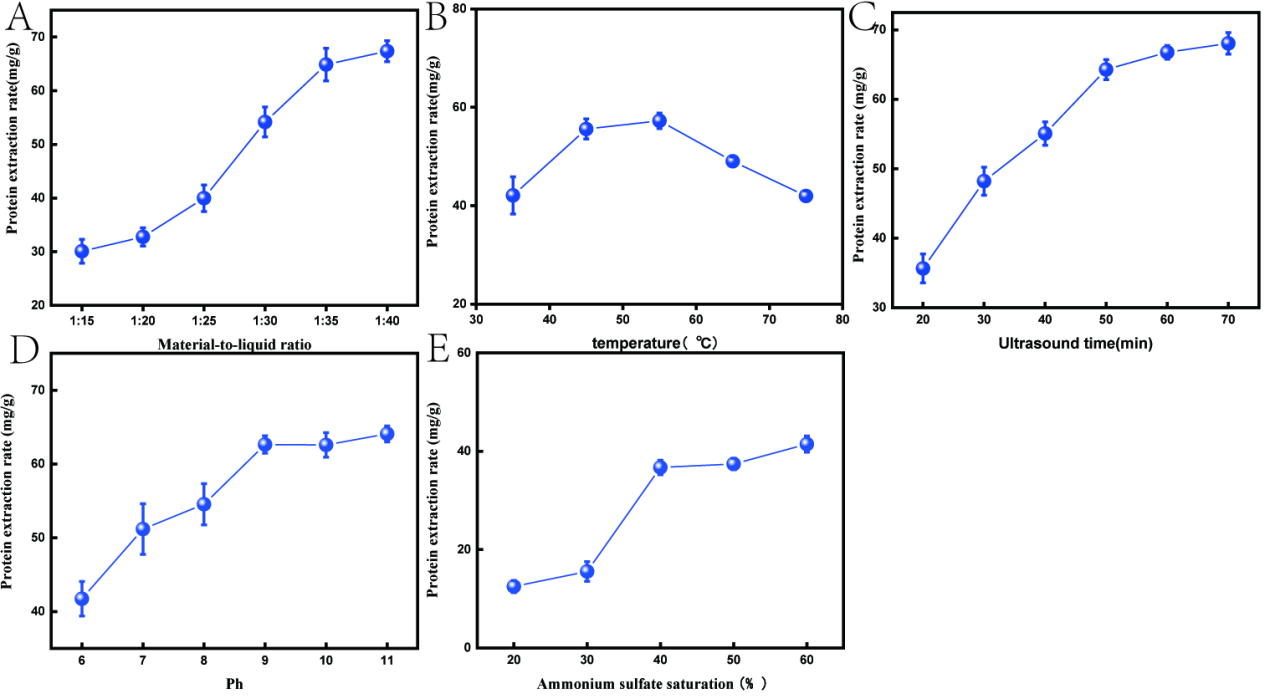


Figure S3 Effect of different conditions on *Moringa oleifera* leaves protein extraction. (A) Material-to-liquid ratio (B) Temperature (C) Ultrasound time (D) pH (E) Ammonium sulfate saturation


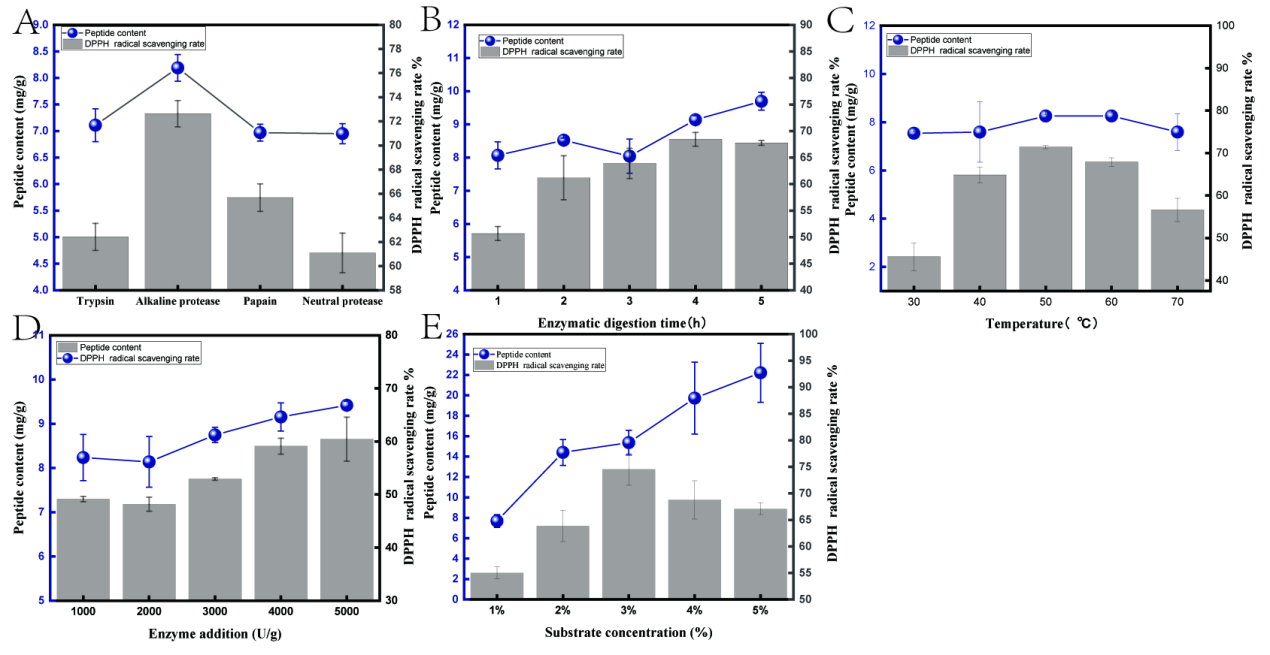


Figure S4 Effect of different conditions on the yield and antioxidant activity of antioxidant peptides from *Moringa oleifera* leaves. (A) Different enzymes (B) Enzymatic digestion time (C) Enzymatic digestion temperature (D) Amount of enzyme added (E) Substrate concentration


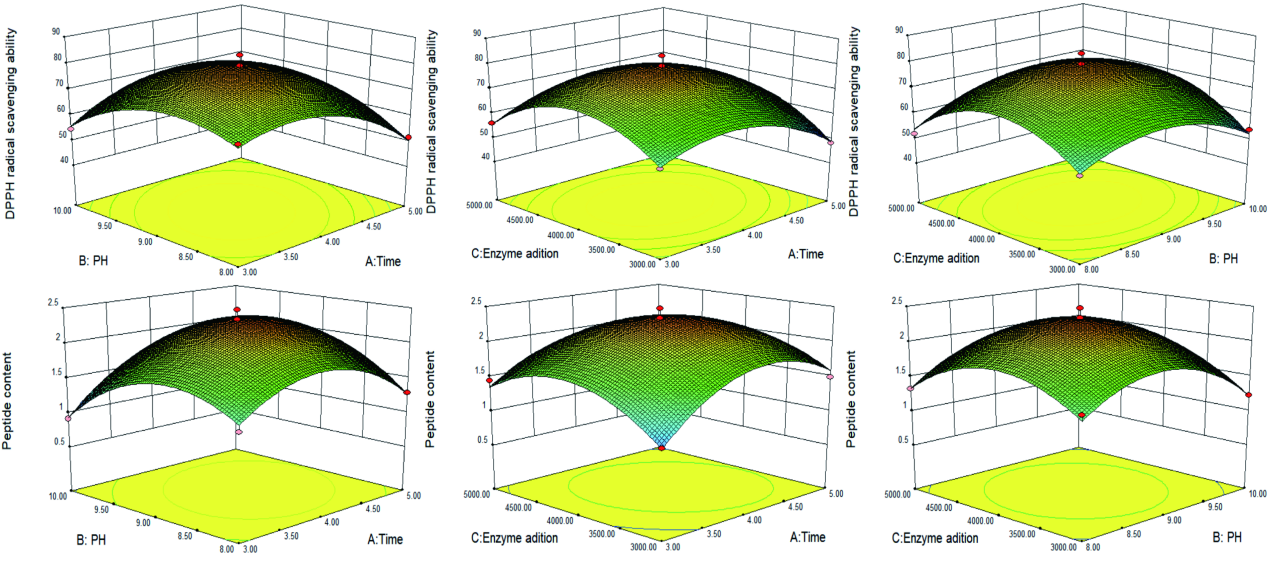


Figure S5 Response surface plots were used to analyze the effects of enzyme addition, enzymatic digestion time and Ph on the scavenging rate of DPPH radicals and peptide content.

Table S1 Results of orthogonal design for *Moringa oleifera* leaves protein extraction

| Test number | A (Temperature/°C) | B (material-liquid ratio/g) | C（pH） | Protein extraction rate/% |
| --- | --- | --- | --- | --- |
| 1 | 1 | 1 | 1 | 61.89 |
| 2 | 1 | 2 | 2 | 54.72 |
| 3 | 1 | 3 | 3 | 78.28 |
| 4 | 2 | 1 | 2 | 59.18 |
| 5 | 2 | 2 | 3 | 66.29 |
| 6 | 2 | 3 | 1 | 54.03 |
| 7 | 3 | 1 | 3 | 58.83 |
| 8 | 3 | 2 | 1 | 59.72 |
| 9 | 3 | 3 | 2 | 68.34 |
| K_1_ | 194.89 | 179.90 | 175.64 |  |
| K_2_ | 192.18 | 180.73 | 181.89 |  |
| K_3_ | 203.75 | 200.65 | 138.00 |  |
| k_1_ | 64.93 | 59.97 | 58.55 |  |
| k_2_ | 64.06 | 60.24 | 60.63 |  |
| k_3_ | 67.91 | 66.88 | 46.00 |  |
| very poor | 3.85 | 6.92 | 14.63 |  |
| Sort by | C>B>A | | | |
| Excellent level | A_3_ | B_3_ | | C_2_ |
| Excellent combination | A_3_B_3_C_2_ | | | |

Table S2 Experimental design and results for response surface analysis

| Number | *A* | *B* | *C* | Peptide content（mg/g） | DPPH free radical scavenging rate（%） |
| --- | --- | --- | --- | --- | --- |
| 1 | 4 | 9 | 4000 | 23.0403 | 83.2813 |
| 2 | 3 | 9 | 5000 | 14.4534 | 56.1553 |
| 3 | 4 | 9 | 4000 | 23.3092 | 75.2217 |
| 4 | 3 | 9 | 3000 | 10.7219 | 57.8923 |
| 5 | 4 | 9 | 4000 | 23.389 | 79.2285 |
| 6 | 5 | 9 | 5000 | 13.6214 | 55.7249 |
| 7 | 5 | 8 | 4000 | 12.8784 | 51.5805 |
| 8 | 5 | 9 | 3000 | 14.9135 | 48.211 |
| 9 | 4 | 8 | 5000 | 13.2863 | 51.9011 |
| 10 | 4 | 8 | 3000 | 15.021 | 55.9633 |
| 11 | 3 | 8 | 4000 | 13.069 | 67.8349 |
| 12 | 3 | 10 | 4000 | 9.163 | 54.4403 |
| 13 | 4 | 9 | 4000 | 24.7199 | 77.3529 |
| 14 | 5 | 10 | 4000 | 16.7542 | 60.7468 |
| 15 | 4 | 10 | 3000 | 12.2581 | 53.913 |
| 16 | 4 | 9 | 4000 | 21.689 | 78.035 |
| 17 | 4 | 10 | 5000 | 13.0659 | 59.757 |

Table S3 DPPH radical scavenging rate and peptide content regression model ANOVA table

| DPPH free radical scavenging rate regression model ANOVA table | | | | | | |  | | | Peptide content regression model ANOVA table | | | | | |
| --- | --- | --- | --- | --- | --- | --- | --- | --- | --- | --- | --- | --- | --- | --- | --- |
| Significance | P-value | F-value | | Mean Square | sum of square |  | | | Sum of square | | Mean Square | | F-value | P-value | Significance |
| ** | < 0.0001 | | 31.74 | 227.5 | 2047.61 | Model | | | 3.90 | | | 0.43 | 32.92 | < 0.0001 | ** |
| * | < 0.0330 | | 7.02 | 50.30 | 50.30 | A（time） | | | 0.14 | | | 0.14 | 11.0 | 0.0128 | * |
|  | 0.8409 | | 0.043 | 0.31 | 0.31 | B(PH） | | | 0.011 | | | 0.011 | 0.86 | 0.3839 |  |
|  | 0.3515 | | 1.00 | 7.14 | 7.14 | C(Enzyme addition amount） | | | 0.00286 | | | 0.00286 | 0.22 | 0.6552 |  |
| ** | 0.0040 | | 17.75 | 127.25 | 127.25 | | AB | 0.15 | | | | 0.15 | 11.51 | 0.0116 | * |
|  | 0.1277 | | 2.98 | 21.39 | 21.39 | | AC | 0.036 | | | | 0.063 | 4.80 | 0.00647 | ** |
|  | 0.1608 | | 3.42 | 24.53 | 24.53 | | BC | 0.016 | | | | 0.016 | 1.23 | 0.33044 |  |
| ** | < 0.0001 | | 63.91 | 458.08 | 458.08 | | A2 | 1.10 | | | | 1.10 | 83.95 | < 0.0001 | ** |
| ** | < 0.0002 | | 53.49 | 383.43 | 383.43 | | B2 | 1.11 | | | | 1.11 | 84.60 | < 0.0001 | ** |
| ** | ＜0.0001 | | 110.21 | 789.99 | 789.99 | | C2 | 0.92 | | | | 0.92 | 70.10 | < 0.0001 | ** |
|  |  | |  | 7.17 | 50.18 | | residual | 0.092 | | | | 0.013 |  |  |  |
|  | 0.6766 | | 0.55 | 4.86 | 14.58 | | lack of fit | 0.045 | | | | 0.015 | 1.99 | 0.2578 |  |
|  |  | |  | 8.90 | 35.59 | | pure error | 0.047 | | | | 0.012 |  |  |  |
|  |  | |  |  | 2097.79 | | comprehensive | 3.99 | | | |  |  |  |  |

Note: *，*P* ＜ 0.05 ； **，*P* ＜ 0.01
