# Supplementary figures and images for: Preparation of antioxidant peptides from Moringa oleifera leaves and their protection against oxidative damage in HepG2 cells
Source: Front Nutr. 2022 Dec 1;9:1062671. doi: 10.3389/fnut.2022.1062671 (PMC9751868; doi:10.3389/fnut.2022.1062671)

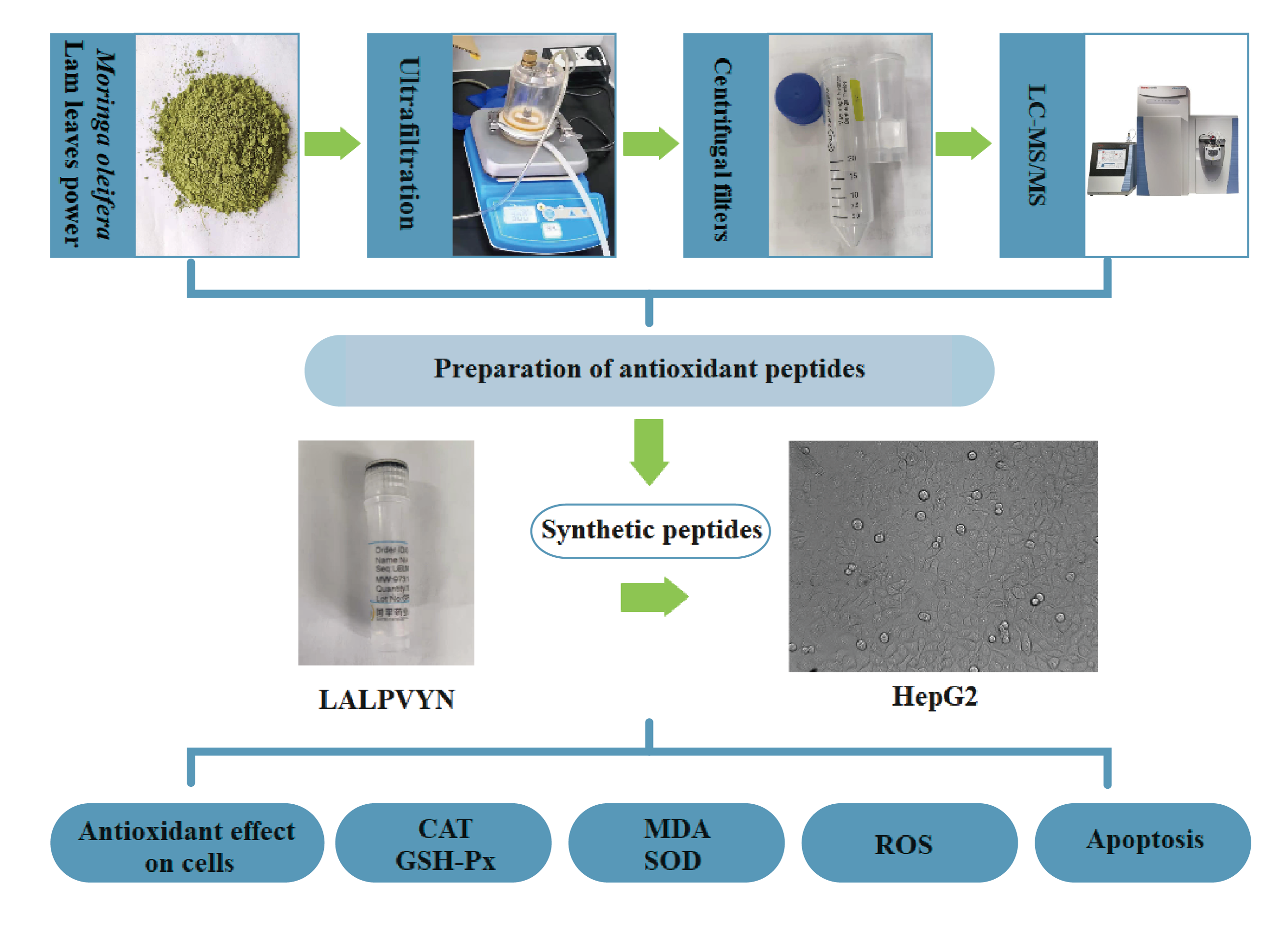

Supplement: Supplementary file 2 [file Image_1.PNG]
